# Impact of multimodal analgesia on postoperative anxiety and depression following total knee arthroplasty

**DOI:** 10.1186/s13018-023-04192-8

**Published:** 2023-09-21

**Authors:** Changjian Zheng, Zijun Hou, Tingting Wang, Lingmei Yi, Yongquan Chen

**Affiliations:** Department of Anesthesiology, The First Affiliated Hospital of Wannan Medical College, 2 Zheshan West Road, Jinghu, Wuhu, 241001 Anhui China

**Keywords:** Multimodal analgesia, Anxiety, Depression, Total knee arthroplasty

## Abstract

**Background:**

Postoperative pain after total knee arthroplasty (TKA) can cause negative emotions, such as anxiety and depression, which can severely affect a patient’s long-term quality of life.

**Objective:**

This study aimed to investigate the impact of multimodal analgesia (MMA) on postoperative anxiety and depression following total knee arthroplasty.

**Methods:**

This study included 161 patients who underwent TKA from October 2020 to October 2022 in the First Affiliated Hospital of Wannan Medical College, including 79 cases in the control group and 82 cases in the multimodal analgesia group (MMA). The MMA group were administered acetaminophen 0.5 g/d orally 3 days before the surgery, and an ultrasound-guided fascia iliac compartment block (FICB) with 0.25% ropivacaine 30 ml in the inguinal region ipsilateral to the surgery was performed 1 h before surgery. After the surgery, 100 ml solution includes 100 mg ropivacaine, 2.5 mg morphine, and 0.25 mg epinephrine for intra-articular and periarticular injection. Postoperative conventional intravenous analgesia was used in the control group, including 100 mg ropivacaine, 2.5 mg morphine, and 0.25 mg epinephrine for intra-articular and periarticular injection. Patients were scored for pain, anxiety, and depression in the ward at 3 and 7 days postoperatively, and postoperative patients were scored using telephone callbacks at 3 months postoperatively.

**Results:**

It was found that the visual analog scale (VAS) scores for pain at rest at 3 days, 7 days, and 3 months postoperatively were significantly lower in the MMA group than in the control group (*P* < 0.05). The scores for pain with movement were significantly lower in the MMA group than in the control group at 3 days and 7 days postoperatively (*P* < 0.01), but they were similar at 3 months postoperatively. Compared to the control group, the MMA group had significantly higher American Knee Society scores (AKS) at 3 days, 7 days, and 3 months postoperatively (*P* < 0.05). Compared to the control group, the MMA group had significantly higher Lower Extremity Functional Scale and Hospital Anxiety and Depression Scale scores (HADS) (*P* < 0.05) at 3 days and 7 days postoperatively; compared to the control group, the MMA group had a significantly shorter hospital stay (*P* < 0.01).

**Conclusion:**

Multimodal analgesia can alleviate postoperative anxiety and depression in the short term, reduce perioperative pain, improve postoperative recovery, and shorten the length of hospital stay.

**Supplementary Information:**

The online version contains supplementary material available at 10.1186/s13018-023-04192-8.

## Introduction

Total knee arthroplasty is a major treatment for severe knee disease, and it can effectively reduce clinical symptoms and improve knee joint function [1]. Typically, TKA patients are elderly and have endured a prolonged preoperative period of pain and suffer from greater postoperative pain [2]. Reportedly, 19%–30% of patients suffer from moderate-to-severe pain following surgery [3]. Pain not only restricts postsurgical functional exercise but also predisposes the patient to negative emotions, such as anxiety and depression [4–6].

Anxiety and depression are believed to be the most common postoperative negative emotions in patients who have undergone TKA [7, 8]. Study found that 7% of patients awaiting TKA had already been diagnosed with anxiety or depression [9]. It has also been reported that 20%–30% of TKA patients suffer from preoperative anxiety and depressive symptoms, and those suspected of having anxiety or depression have a poorer prognosis than those in a better mental state [10, 11]. Preoperative anxiety, depression, pain, and dysfunction are major predictors of adverse postoperative health-related effects on a patient’s quality of life [12]. It has been proved that anxiety changes the sensitivity to pain, i.e., increased anxiety is associated with increased sensitivity to pain [13]. Negative emotions are significantly improved in most patients undergoing surgery if their postoperative pain is effectively alleviated and functional mobility restored [14]. The above studies all showed that pain is a leading cause of postoperative negative emotions, including anxiety and depression.

Multimodal analgesia, which combines analgesics that have different mechanisms and uses a range of methods to achieve balanced analgesia, has become a widely recognized analgesic method. The combined analgesics exert a synergistic or additive analgesic effect, and using them in combination reduces the dose and the adverse reactions associated with a single analgesic. Multimodal analgesia has been found to significantly reduce the VAS score and the side effects of nausea and vomiting and alleviates anxiety symptoms in patients following TKA, thereby contributing to their early recovery [15]. The present study aimed to explore the impact of analgesia achieved by an MMA intervention upon patients’ postoperative anxiety and depression scores, with a view to discovering a way to improve their postoperative quality of life.

## Methods

### Ethical approval and patient eligibility criteria

**Fig. 1:**
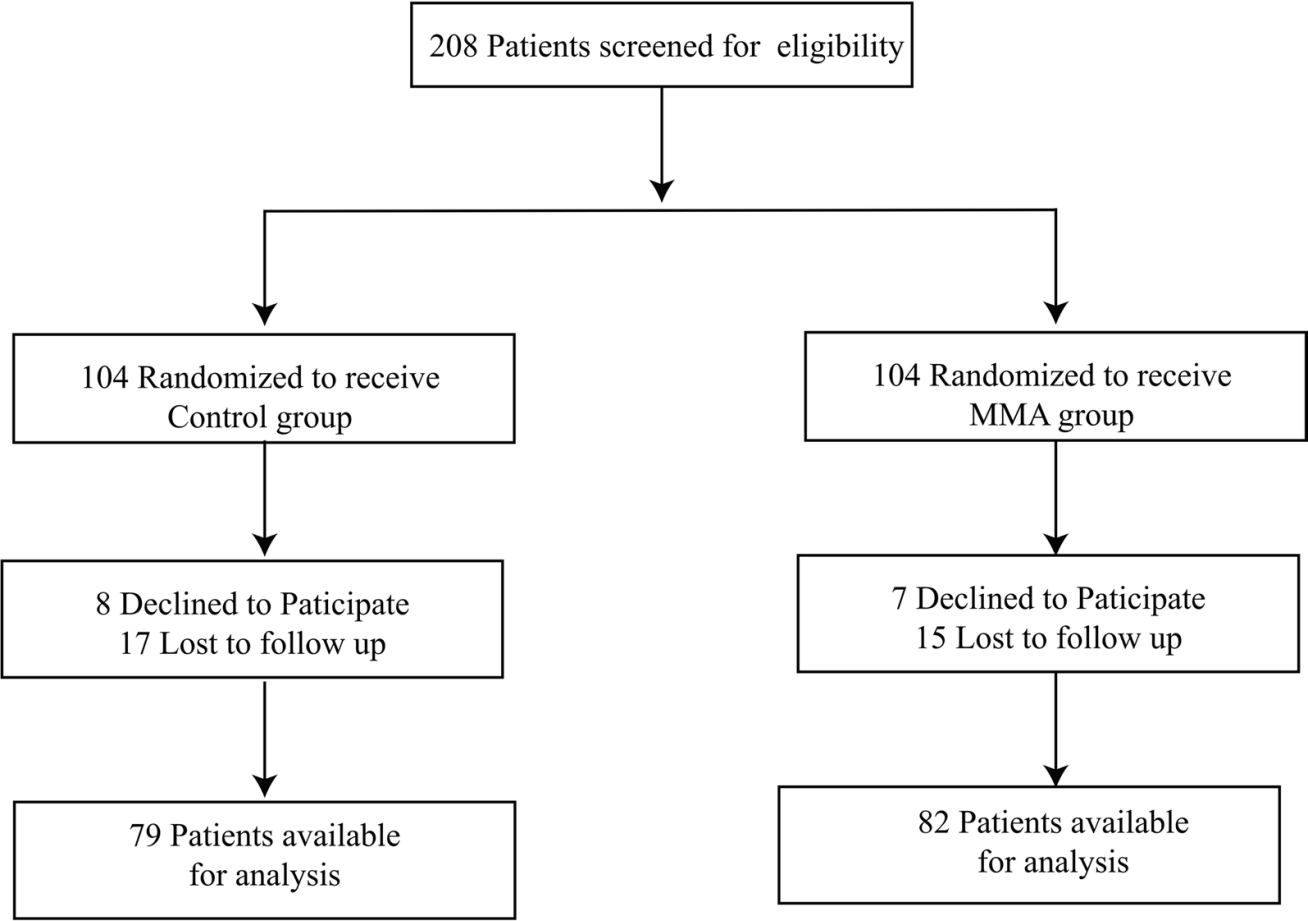
Patient registration begins on October 1, 2020, and ends on October 1, 2022. A CONSORT flow chart is given in Fig. 1

This study was approved by the Ethics Committee of the First Affiliated Hospital of Wannan Medical College, which assented to the collection of data from patients who underwent a unilateral TKA between October 2020 and October 2022 at the First Affiliated Hospital of Wannan Medical College, Wuhu, China. All the patients and/or their relatives provided written informed consent. The patients were divided into two groups using the computerized random order method, and postoperative follow-up was performed without the clinician knowing the specific analgesic implementation plan of the individual patient. A total of 208 patients were randomly enrolled in this study initially and assigned to the control group and the MMA group (Fig. 1). The patients were 54–85 years old, had a body mass of 45–90 kg, and were American Society of Anesthesiologists (ASA) class I–III. However, 15 patients were lost during the follow-up, and 32 patients met the exclusion criteria, which were as follows: (1) patients with a communication disability who were unable to complete a questionnaire; (2) patients who had a definitive mental disorder or were taking medication for such a disorder; (3) patients with any recent significant psychological issues; or (4) patients with cognitive dysfunction postoperatively.

### Ultrasound-guided fascia iliaca compartment block

**Fig. 2:**
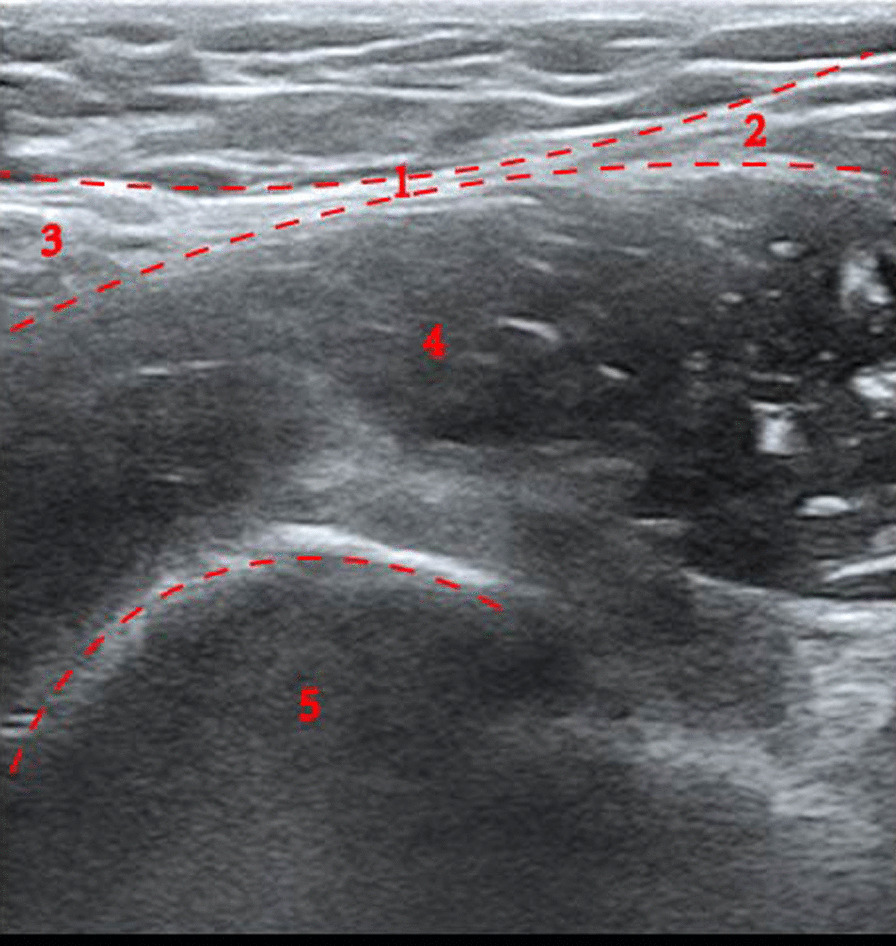
Fascia iliaca compartment block: bow-tie in the iliac fascia region on ultrasound. 1. Bow tie sign 2. Sartorius muscle 3. Internal oblique muscle 4. Iliac muscle 5. Anterior superior iliac spine

The patient was sent to the operating theater 1 h before the surgery, and routine peripheral venous access was established. An ultrasound-guided fascia iliaca compartment block (FICB) was performed under electrocardiographic monitoring. After routine disinfection and draping, a high-frequency linear-array transducer was placed vertically onto the groin region and parallel to the inguinal ligament. After the femoral artery was identified, the transducer was slid 90° counterclockwise and moved cranially to the bow–tie structure, which lies laterally to the anterior superior iliac spine at about 1/3rd the distance from the pubic tubercle. The needle penetrated through the fascia lata, reaching beneath the fascia iliaca, and delivered a local anesthetic injection of 30 mL containing 0.25% ropivacaine. The diffusion of local anesthetic was observed under ultrasound, while cold sensation was tested on the blocked and nonblocked sides using a cotton ball moistened with 75% alcohol until the patient perceived that there was a significant difference in cold sensation between the two sides, indicating that the block was successful (Fig. 2).

### Intra-articular and periarticular injection

**Fig. 3:**
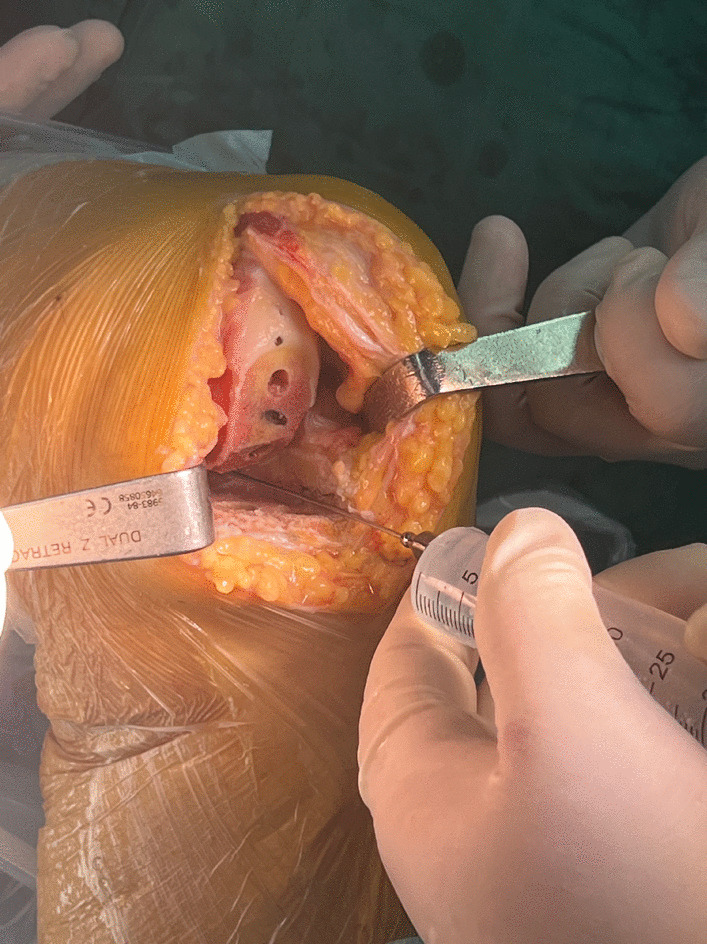
Local anesthetic injection in the joint cavity and around the joint

A 100-mL volume of solution containing 100 mg ropivacaine, 2.5 mg morphine, and 0.25 mg epinephrine in normal saline was prepared. Prior to prosthesis implantation, the first 1/3rd of the solution was injected into the posterior knee joint capsule, followed by the posterolateral and medial aspects of the knee joint. When the bone cement solidified, the second 1/3rd third was injected near the quadriceps, and the last 1/3rd was injected into the fat and subcutaneous tissue (Fig. 3).

### Anesthesia regimen

All the patients were asked to fast for 4–6 h before the surgery. Routine peripheral venous access was established, and the patient’s heart rate, blood pressure, and oxygen saturation of arterial blood were monitored (Spacelabs monitor, USA). To maintain appropriate anesthesia depth, bispectral index (BIS) monitoring (Covidien IIc Co., USA) was utilized, The BIS was targeted to be maintained between 40 and 60. During the induction of the anesthesia, midazolam (0.02 mg/kg), propofol (2 mg/kg), sufentanil (0.4 g/kg), and cis-atracurium (0.2 mg/kg) were administered to the patients in both groups. Endotracheal intubation was performed using a 7.0 or 7.5 endotracheal tube after the muscle relaxants took effect. The anesthesia was maintained intraoperatively by administering propofol (4–6 mg/kg/h) and remifentanil (0.01–0.02 g/kg/min).

A routine analgesia regimen was employed in the control group, with 100 mL sufentanil 2 g/kg via a patient-controlled analgesia pump at a background infusion rate of 2 mL/h, a bolus dose of 2 mL, and a lockout interval of 30 min. In the MMA group, acetaminophen (0.5 g/d) was administered 3 days before the surgery, and ultrasound-guided FICB was applied at the surgery site 1 h. before surgery. Subsequently, intra-articular and periarticular injections and routine analgesia were administered in a similar manner to the control group. All procedures were performed by the same joint surgeon.

Baseline data, such as height, body mass index (BMI), and sex, were collected from both groups. HADS (score 0–7: normal level. Scores of 8–10: mild anxiety and depression. Score 11–21: severe anxiety and depression), AKS (total score 0–100, with higher scores indicating better status), and VAS scores were used to evaluate the patients the day before the TKA, as well as 3 days, 7 days, and 3 months postoperatively.

### Statistical analysis and sample size estimation

We used G-Power software for sample size estimation. This study was a randomized controlled trial with two groups, control group and MMA group, where patients' HADS score was the primary outcome. According to our preliminary pilot study, the HADS scores of the control group were 14.5 ± 4.6, respectively. The HADS scores of the MMA group were 12.8 ± 3.0, The power was set at 80% with two-sided ɑ = 0.05. According to the calculation, the total sample size was obtained as 166 cases, and the final calculation considering 20% of lost and refused visits, the total number of study patients needed was 200, of which at least 100 patients were needed in each group. We ultimately enrolled 208 patients.

The statistical analysis was performed using SPSS26.0 software and GraphPad Prism 8. Measurement data were expressed as mean ± standard derivation (mean ± SD). Enumeration data were analyzed using a χ^2^ test, and the measurement data of the two groups were compared using an independent t test; *P* < 0.05 indicated a statistically significant difference.

## Results

### Comparison of baseline data

No significant difference was noted between the two groups with respect to age, sex, history of chronic disease, BMI, operating time, anesthetic dosage, hemodynamics, preoperative VAS score at rest, preoperative VAS score with movement, HADS anxiety score, HADS depression score, or AKS score (all *P* > 0.05) (Additional file 1, 2 and 3). However, the MMA group had a significantly shorter hospital stay than the control group (*P* < 0.01, Table 1).

**Table 1:** Comparison of patients’ baseline data between the two groups

Index	Control group (n = 79)	MMA group (n = 82)	Statistical value	*P*
Age (years)	67 ± 10	65 ± 10	*t* = 1.588	0.114
Sex (male/female, n)	31/48	43/39	χ^2^ = 0.450	0.518
History of chronic disease (yes/no, n)	42/37	43/39	χ^2^ = 0.008	1.000
Body mass index ( kg/m^2^)	23.5 ± 2.8	23.8 ± 2.4	*t* = 0.683	0.489
Operating time (min)	101.7 ± 27.0	98.9 ± 31.8	*t* = 0.611	0.542
Preoperative VAS score at rest	1.8 ± 1.3	1.5 ± 1.1	*t* = 1.438	0.152
Preoperative VAS score at movement	5.6 ± 2.1	5.3 ± 1.7	*t* = 1.053	0.294
Preoperative HAD anxiety score	8.1 ± 2.6	8.4 ± 2.6	*t* = 0.763	0.447
Preoperative HAD depression score	9.4 ± 3.1	10.2 ± 3.2	*t* = 1.408	0.161
Preoperative AKS score	56.0 ± 15.0	52.8 ± 11.9	*t* = 1.477	0.142
Hospital stay ( d)	12 ± 2	10 ± 3^▲^	t = 3.084	0.002

The data are reported as mean ± SD or the number of subjects

Compared to the control group, ^▲^*P* < 0.001

### Visual analog scale scores

Compared to the control group, the MMA group showed significantly lower scores at rest at 3 days, 7 days, and 3 months postoperatively (*P* < 0.05 or *P* < 0.01). The VAS scores with movement were significantly lower in the MMA group than the control group at 3 days and 7 days postoperatively (*P* < 0.01) but were similar at 3 months postoperatively (*P* > 0.05, Table 2).

**Table 2:** Comparison of VAS scores at rest and movement between patients from the two groups

Group	VAS score at rest				VAS score at movement			
	Preoperative	3 days postoperatively	7 days postoperatively	3 months postoperatively	Preoperative	3 days postoperatively	7 days postoperatively	3 months postoperatively
Control group	1.8 ± 1.3	6.0 ± 2.1	2.9 ± 1.4	1.5 ± 0.7	5.6 ± 2.1	7.7 ± 1.3	6.1 ± 1.8	2.2 ± 1.0
MMA group	1.5 ± 1.1	4.8 ± 2.0^▲^	2.0 ± 0.9^▲^	1.3 ± 0.7^**★**^	5.3 ± 1.7^▲^	6.6 ± 1.9^▲^	5.3 ± 2.0^**★**^	1.9 ± 0.7
*t* value	1.438	3.606	4.623	2.10	1.053	4.034	2.416	1.488
*P*	0.152	< 0.01	< 0.01	< 0.05	0.294	< 0.01	< 0.05	0.139

The data are reported as means ± SD, The t test is used

Compared to the control group, ^**★**^*P* < 0.001, ^▲^*P* < 0.01

### American Knee Society scores

Compared to the control group, the MMA group had significantly higher AKS scores at 3 days, 7 days, and 3 months postoperatively (*P* < 0.01, Table 3).

**Table 3:** Comparison of AKS scores between patients from the two groups

Group	AKS score			
	Preoperative	3 days postoperatively	7 days postoperatively	3 months postoperatively
Control group	56.0 ± 15.0	41.9 ± 9.5	62.2 ± 11.0	90.1 ± 12.4
MMA group	52.8 ± 11.9	49.3 ± 14.2^▲^	71.5 ± 14.0^▲^	96.3 ± 14.3^▲^
*t* value	1.447	3.842	4.676	2.911
*P*	0.142	< 0.01	< 0.01	< 0.01

The data are reported as means ± SD. The t test is used

Compared to the control group, *P*^▲^ < 0.01

### Hospital Anxiety and Depression Scale scores

Patients from both groups suffered from mild anxiety and depression before surgery, but the difference in the HADS scores was not significant (*P* > 0.05). At 3 and 7 days postoperatively, the HADS scores were significantly lower in the MMA group than in the control group (*P* < 0.01). No difference was noted between the two groups at 3 months postoperatively (*P* > 0.05, Table 4).

**Table 4:** Comparison of HAD scores between patients from the two groups

Group	HAD score							
	Preoperative		3 days postoperatively		7 days postoperatively		3 months postoperatively	
	Anxiety score	Depression score	Anxiety score	Depression score	Anxiety score	Depression score	Anxiety score	Depression score
Control group	8.1 ± 2.6	9.4 ± 3.1	13.2 ± 5.3	13.2 ± 3.5	11.8 ± 4.7	10.4 ± 3.7	5.5 ± 2.1	5.5 ± 2.0
MMA group	8.4 ± 2.6	10.2 ± 3.2	10.7 ± 4.6^▲^	11.2 ± 2.8^▲^	9.2 ± 3.4^▲^	8.4 ± 2.8^▲^	5.1 ± 1.8	5.1 ± 1.8
*t* value	0.763	1.408	3.141	4.027	4.023	3.951	1.209	1.264
*P*	0.447	0.161	< 0.01	< 0.01	< 0.01	< 0.01	0.229	0.208

The data are reported as means ± SD. The t test is used

Compared to the control group, ^▲^*P* < 0.01

## Discussion

Total knee arthroplasty has become the first choice of treatment for advanced knee osteoarthritis [16]_._ However, the severe perioperative pain associated with TKA brings great suffering to patients, predisposing them to negative emotions, such as anxiety and depression [17]. Therefore, effective analgesia is crucial to the functional recovery of the knee joint following TKA.

Studies show analgesia after TKA is often poor. Severe postoperative pain makes patients reluctant to do early postoperative rehabilitation or functional exercises, which might result in suboptimal functional restoration of the knee joint [3], significantly decreased postoperative satisfaction, and a poorer quality of life. In one postoperative survey, about 20% of the patients expressed dissatisfaction, which predisposed them to negative emotions, which might have affected their recovery [18]_._

No consensus has yet been reached regarding the standardized analgesia management of TKA. Presently, nonsteroidal drugs, nerve block, intra-articular injection, and other multimodal methods are recommended pre-, intra-, and postoperatively to increase the analgesic effect and reduce the use of opioids. In order to reduce the adverse effects caused by preoperative pain, the present study adopted the concept of preventive analgesia and administered oral medication with nonsteroidal anti-inflammatory agent acetaminophen 3 days prior to the surgery. Reportedly, the administration of nonsteroidal agents significantly reduces the postoperative VAS score, and therefore, nonsteroidal agents serve as a safe and effective perioperative choice for significantly improving analgesia following TKA [19]_._

The fascia iliac compartment is a space that is restricted anteriorly by the fascia iliaca and posteriorly by the iliacus and psoas major muscles. The femoral nerve shares a common passage with the lateral femoral cutaneous nerve, obturator nerve, and femoral branch of the genitofemoral nerve posterior to the fascia iliaca. Fascia iliaca compartment block is a low-skill technique that has little risk of causing neurovascular injury because the entry point of the injection is distal to the neurovascular sheath. The technique has been increasingly applied in the analgesia management of TKA, and a previous study showed that ultrasound-guided FICB reduces the usage of postoperative morphine 24 h after TKA [20]_._ Classic ultrasound-guided FICB places the transducer parallel to the inguinal ligament and adopts an in-plane approach for the distal puncture injection, so the drugs cannot diffuse to the head, which results in an incomplete block [21, 22]_._ Swenson et al. used magnetic resonance imaging to observe anesthetic distribution during the classic ultrasound-guided FICB [23]_._ The study speculated that the injectate failed to reach the pectineus muscle and, thus, could not block the obturator nerve. The present study used a modified FICB that placed the transducer vertically onto the groin region and parallel to the inguinal ligament. After the femoral artery was identified, the hourglass pattern was used to search for the bow tie. Hebbard et al. [24] used a modified FICB in > 150 patients and achieved excellent outcomes, while other studies have demonstrated that the hourglass pattern might help in recognizing the fascia iliaca, simplify the FICB technique, and increase its safety [25, 26]. Kumar et al. compared the conventional FICB and the modified proximal supra-inguinal FICB for postoperative analgesia in TKA and found significantly less morphine consumption and lower VAS scores in patients accepting the modified FICB [27]. Thus, this study adopted the modified ultrasound-guided FICB for postoperative analgesia.

Theoretically, the modified FICB obstructs the nerves innervating the knee joint and the surrounding tissues. However, these nerves are a complex network, and the sacral plexus and its branches also innervate the knee joint capsule and the surrounding tissues (although anatomical variations of the cutaneous branch of the obturator nerve are not uncommon), often causing incomplete analgesia [23]. Thus, other adjuvant analgesia methods are still needed. Local infiltration anesthesia with an analgesic cocktail is a novel analgesic for TKA and has certain advantages. The injection can be given under direct vision and is easy to administer, and the analgesic spreads straight to the desired site and has fewer adverse effects. Nakai et al. found that periarticular injection with multimodal analgesics can significantly reduce postoperative pain [28]_._ Fajardo et al. also found that cocktail analgesia could effectively control postoperative pain following TKA [29]_._ Other studies have also found that cocktail analgesia significantly alleviates postoperative pain and shortens hospital stays without affecting wound healing [30, 31]. Accordingly, this study used cocktail analgesia as an adjunct to the nerve block to achieve the best analgesic regimen.

One large-sample meta-analysis showed a steadily increasing prevalence of psychiatric disorders in TKA patients, with anxiety and depression accounting for 13.29% of the observed disorders [32]. Other studies have shown that anxiety and depression are present in TKA patients preoperatively, with a prevalence of 20.2%–38% [33, 34]. There is a growing body of evidence implicating psychosocial factors, including anxiety, depression, pain catastrophizing, as negative prognostic factors following total knee arthroplasty (TKA) [7]. Postoperative pain makes the patient fearful of postoperative functional rehabilitation and jeopardizes functional restoration. Anxiety and depression are the psychological symptoms correlated with arthroplasty and prognosis in patients with osteoarthritis and are among the most common complications of chronic pain. Daniel et al. conducted a 6-month follow-up of 140 TKA patients. Their study found that preoperative psychological predictors affected the restoration of the knee function (but with no significant correlation), thereby suggesting the need for a rational psychological intervention focusing on preoperative pain catastrophizing [35]. Associated studies have indicated that anxiety and depression affect analgesia and the function of the knee joint, while mutual interactions exist between mood changes and pain. Therefore, the emotions of most patients undergoing surgery should improve significantly after optimal analgesia is achieved [14]. The current study observed the presence of negative emotions, such as anxiety and depression, in TKA patients preoperatively, but these emotions improved significantly after the alleviation of postoperative pain and increased range of movement.

This study divided the MMA into a 3-phase regimen consisting of pre-, intra-, and postoperative analgesia. Thus, preventive analgesia, ultrasound-guided FICB, and support from the surgeons were merged into an MMA regimen. During the study, data were collected from all the patients, and their VAS, AKS lower limb function, and HADS scores were assessed via preoperative clinic visits and questionnaire surveys both preoperatively and at 3 days, 7 days, and 3 months postoperatively. The aim was to explore whether MMA could reduce perioperative pain, improve anxiety and depression, and accelerate rehabilitation. The results showed statistical differences in the HADS scores between the two groups of patients at 3 and 7 days postoperatively, indicating that MMA could effectively improve the patients’ anxiety and depression in the short term. Duivenvoorden et al. prospectively investigated 133 TKA patients and found that the relief of postoperative pain was associated with improved anxiety and depression symptoms [10]. Hassett et al*. *[36] explored whether anxiety and depression improved with pain relief in patients and also found that the postoperative decrease in pain was associated with reduced levels of depression and anxiety after surgery. The present study found that MMA relieves postoperative pain; the mean VAS scores at rest were significantly lower in the MMA group than the control group at all time points, while the mean VAS scores with movement were significantly lower in the MMA group than the control group at 3 and 7 days postoperatively. This phenomenon indicates that MMA relieves perioperative pain in patients, which is consistent with the findings of a previous study [37]. In addition, the AKS scores were higher in the MMA group than in the control group, which indirectly suggests a rapid rehabilitation rate in the MMA patients. This was also similar to previous reports [38, 39]_._

The present study did not find any significant difference in the VAS scores with movement or the HADS scores of the two groups at 3 months postoperatively. This might be attributed to the rapid recovery rate and low incidence of long-term negative emotions in younger patients. However, the patients were not stratified by age, and the sample size was small, so more in-depth investigation is required in future studies. 

## Conclusion

The results of this study indicate that MMA alleviates postoperative anxiety and depression in the short term, reduces perioperative pain, improves postoperative recovery, and shortens the length of hospital stay for patients undergoing TKA.

### Supplementary Information

**Additional file 1:** Comparison of anesthetic drugs and hemodynamics between the two groups.

**Additional file 2:** American Knee Society Knee Score(AKS) Assessment Scale.

**Additional file 3:** Hospital Anxiety and Depression Scale (HADS) Assessment Scale.

## Data Availability

The datasets used and/or analyzed during the current study are available from the corresponding author on reasonable request.

## Abbreviations

TKA: Total knee arthroplasty
MMA: Multimodal analgesia
VAS: Visual analog scale
FIC: Fascia iliaca compartment
FICB: Fascia iliaca compartment block
BMI: Body mass index
HAD: Hospital Anxiety and Depression Scale
AKS: American Knee Society score
ASA: American Society of Anesthesiologist
