# Supplementary material for: Impact of multimodal analgesia on postoperative anxiety and depression following total knee arthroplasty
Source: J Orthop Surg Res. 2023 Sep 21;18:712. doi: 10.1186/s13018-023-04192-8 (PMC10515006; doi:10.1186/s13018-023-04192-8)
Supplement: Supplementary file 1 — Additional file 1: Comparison of anesthetic drugs and hemodynamics between the two groups. [file 13018_2023_4192_MOESM1_ESM.doc]

**Supplement Table** Comparison of anesthetic drugs and hemodynamics between the two groups

| Index | Control group  (n=79) | MMA group  (n=82) | Statistical value | *P* |
| --- | --- | --- | --- | --- |
| MAP (mmHg) | 88±9 | 90±10 | t=1.342 | 0.182 |
| HR (beat/min) | 74±10 | 76±10 | t=1.274 | 0.205 |
| Basic BIS | 85±7 | 84±7 | t=1.250 | 0.213 |
| Propofol (mg) | 770±133 | 795±110 | t=1.289 | 0.199 |
| Remifentanil (ug) | 1531±360 | 1571±309 | t=0.745 | 0.458 |
| Cis-Atracurium(mg) | 10.8±2.2 | 11.0±1.8 | t=0.454 | 0.650 |
| Sufentanil(ug) | 25.3±6.1 | 25.7±4.9 | *t*=0.547 | 0.585 |

MAP:mean arterial pressure, HR:Heart beat, BIS:bispectral index

The data are reported as mean ± SD or the number of subjects
