# Supplementary figures and images for: Impact of multimodal analgesia on postoperative anxiety and depression following total knee arthroplasty
Source: J Orthop Surg Res. 2023 Sep 21;18:712. doi: 10.1186/s13018-023-04192-8 (PMC10515006; doi:10.1186/s13018-023-04192-8)

**American Knee Society Knee Score(AKS)**


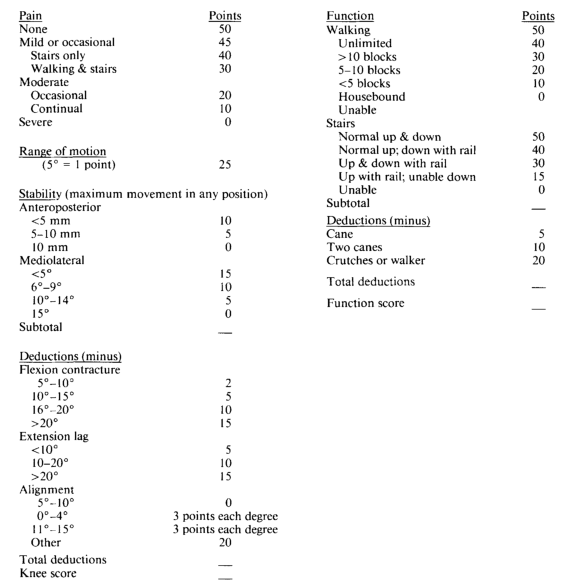

Supplement: Supplementary file 2 — Additional file 2: American Knee Society Knee Score(AKS) Assessment Scale. [file 13018_2023_4192_MOESM2_ESM.doc]
