# Supplementary material for: Impact of multimodal analgesia on postoperative anxiety and depression following total knee arthroplasty
Source: J Orthop Surg Res. 2023 Sep 21;18:712. doi: 10.1186/s13018-023-04192-8 (PMC10515006; doi:10.1186/s13018-023-04192-8)
Supplement: Supplementary file 3 — Additional file 3: Hospital Anxiety and Depression Scale (HADS) Assessment Scale. [file 13018_2023_4192_MOESM3_ESM.doc]

**Hospital Anxiety and Depression Scale (HADS)**

| A | **I feel tense or 'wound up':** |  |
| --- | --- | --- |
|  | Most of the time | 3 |
|  | A lot of the time | 2 |
|  | From time to time, occasionally | 1 |
|  | Not at all | 0 |

| D | **I still enjoy the things I used to enjoy:** |  |
| --- | --- | --- |
|  | Definitely as much | 0 |
|  | Not quite so much | 1 |
|  | Only a little | 2 |
|  | Hardly at all | 3 |

| A | **I get a sort of frightened feeling as if something awful is about to happen:** |  |
| --- | --- | --- |
|  | Very definitely and quite badly | 3 |
|  | Yes, but not too badly | 2 |
|  | A little, but it doesn't worry me | 1 |
|  | Not at all | 0 |

| D | **I can laugh and see the funny side of things:** |  |
| --- | --- | --- |
|  | As much as I always could | 0 |
|  | Not quite so much now | 1 |
|  | Definitely not so much now | 2 |
|  | Not at all | 3 |

| A | **Worrying thoughts go through my mind:** |  |
| --- | --- | --- |
|  | A great deal of the time | 3 |
|  | A lot of the time | 2 |
|  | From time to time, but not too often | 1 |
|  | Only occasionally | 0 |

| D | **I feel cheerful:** |  |
| --- | --- | --- |
|  | Not at all | 3 |
|  | Not often | 2 |
|  | Sometimes | 1 |
|  | Most of the time | 0 |

| A | **I can sit at ease and feel relaxed:** |  |
| --- | --- | --- |
|  | Definitely | 0 |
|  | Usually | 1 |
|  | Not Often | 2 |
|  | Not at all | 3 |

| D | **I feel as if I am slowed down:** |  |
| --- | --- | --- |
|  | Nearly all the time | 3 |
|  | Very often | 2 |
|  | Sometimes | 1 |
|  | Not at all | 0 |

| A | **I get a sort of frightened feeling like 'butterflies' in the stomach:** |  |
| --- | --- | --- |
|  | Not at all | 0 |
|  | Occasionally | 1 |
|  | Quite Often | 2 |
|  | Very Often | 3 |

| D | **I have lost interest in my appearance:** |  |
| --- | --- | --- |
|  | Definitely | 3 |
|  | I don't take as much care as I should | 2 |
|  | I may not take quite as much care | 1 |
|  | I take just as much care as ever | 0 |

| A | **I feel restless as I have to be on the move:** |  |
| --- | --- | --- |
|  | Very much indeed | 3 |
|  | Quite a lot | 2 |
|  | Not very much | 1 |
|  | Not at all | 0 |

| D | **I look forward with enjoyment to things:** |  |
| --- | --- | --- |
|  | As much as I ever did | 0 |
|  | Rather less than I used to | 1 |
|  | Definitely less than I used to | 2 |
|  | Hardly at all | 3 |

| A | **I get sudden feelings of panic:** |  |
| --- | --- | --- |
|  | Very often indeed | 3 |
|  | Quite often | 2 |
|  | Not very often | 1 |
|  | Not at all | 0 |

| D | **I can enjoy a good book or radio or TV program:** |  |
| --- | --- | --- |
|  | Often | 0 |
|  | Sometimes | 1 |
|  | Not often | 2 |
|  | Very seldom | 3 |

|  | Scoring (add the As = Anxiety.  Add the Ds = Depression).  The norms below will give you an idea of the level of Anxiety and Depression. |  |
| --- | --- | --- |
|  | 0-7 = Normal |  |
|  | 8-10 = Borderline abnormal |  |
|  | 11-21 = Abnormal |  |
|  |  |  |
